# A 10-year follow‐up on the practice of luteal phase support using worldwide web‐based surveys

**DOI:** 10.1186/s12958-021-00696-2

**Published:** 2021-01-26

**Authors:** Gon Shoham, Milton Leong, Ariel Weissman

**Affiliations:** 1 Sackler Faculty of Medicine, Tel Aviv University, Ramat Aviv, P.O.B. 39040, 69978 Tel Aviv, Israel; 2 The IVF Clinic, 13/F Central Tower, 28 Queens Road Central, Hong Kong, China; 3 IVF Unit, Department of Obstetrics and Gynecology, Edith Wolfson Medical Center, 62 Halochamim Street, 5822012 Holon, Israel

**Keywords:** IVF, Luteal phase support, Survey, Progesterone

## Abstract

**Background:**

It has been demonstrated that luteal phase support (LPS) is crucial in filling the gap between the disappearance of exogenously administered hCG for ovulation triggering and the initiation of secretion of endogenous hCG from the implanting conceptus. LPS has a pivotal role of in establishing and maintaining in vitro fertilization (IVF) pregnancies. Over the last decade, a plethora of studies bringing new information on many aspects of LPS have been published. Due to lack of consent between researchers and a dearth of robust evidence-based guidelines, we wanted to make the leap from the bench to the bedside, what are the common LPS practices in fresh IVF cycles compared to current evidence and guidelines? How has expert opinion changed over 10 years in light of recent literature?

**Methods:**

Over a decade (2009–2019), we conducted 4 web-based surveys on a large IVF-specialist website on common LPS practices and controversies. The self-report, multiple-choice surveys quantified results by annual IVF cycles.

**Results:**

On average, 303 IVF units responded to each survey, representing, on average, 231,000 annual IVF cycles. Most respondents in 2019 initiated LPS on the day of, or the day after egg collection (48.7 % and 36.3 %, respectively). In 2018, 72 % of respondents administered LPS for 8–10 gestational weeks, while in 2019, 65 % continued LPS until 10–12 weeks. Vaginal progesterone is the predominant delivery route; its utilization rose from 64 % of cycles in 2009 to 74.1 % in 2019. Oral P use has remained negligible; a slight increase to 2.9 % in 2019 likely reflects dydrogesterone’s introduction into practice. E2 and GnRH agonists are rarely used for LPS, as is hCG alone, limited by its associated risk of ovarian hyperstimulation syndrome (OHSS).

**Conclusions:**

Our Assisted reproductive technology (ART)-community survey series gave us insights into physician views on using progesterone for LPS. Despite extensive research and numerous publications, evidence quality and recommendation levels are surprisingly low for most topics. Clinical guidelines use mostly low-quality evidence. There is no single accepted LPS protocol. Our study highlights the gaps between science and practice and the need for further LPS research, with an emphasis on treatment individualization.

## Key message

This longitudinal survey reveals that trends in the practice of luteal phase support have remained practically unchanged over a 10-year period, and shows a considerable gap between practice and science. Despite extensive research, the quality of evidence remains low for most luteal phase support topics, which emphasizes the need for further research.

## Introduction

During the early phases of spontaneous human pregnancies, the corpus luteum (CL) graviditatis supports the developing conceptus up to the establishment of the utero-placental shift, at around 8 gestational weeks. In assisted reproductive technology (ART) cycles, the fine balance of controlling normal CL function is severely disrupted, despite the presence of multiple corpora lutea, resulting in a deficient luteal phase that must be pharmaceutically supported [1]. Early studies have estimated that exogenously administered hCG remains in circulation for up to 7 days [2], and that the CL has a remarkable ability to recover after prolonged deprivation of gonadotropin stimulation [3]. It has been clearly demonstrated that luteal phase support (LPS) is crucial in filling the gap between the disappearance of exogenously administered hCG for ovulation triggering and the initiation of secretion of endogenous hCG from the implanting conceptus. Thus, the pivotal role of LPS in establishing and maintaining in vitro fertilization (IVF) pregnancies has been one of the earliest subjects to become evidence-based in clinical ART[4], and controlled ovarian stimulation (COS) per se constitutes an indication for LPS [5].

While the use of LPS following IVF/ intracytoplasmic sperm injection (ICSI) is widely accepted, and progesterone (P) is strongly recommended as its major constituent [6], there is no standard available regarding the formulation and route of administration, or on the timing and treatment duration. Moreover, the combination of medications that should be included in LPS regimens, and whether and how we should individualize LPS and tailor treatment to each patient has not been fully established either.

Due to lack of consent between researchers and a dearth of robust evidence-based guidelines, we wanted to make the leap from the bench to the bedside, and to learn directly from the clinicians what the prevailing practices are in the “real world” regarding LPS. Although systematic reviews and meta-analyses constitute the highest level of evidence, results from the most recent version of the Cochrane Collaboration report on LPS remain inconclusive for many of the issues raised [7]. The authors comment that the main limitations in the evidence were poor reporting of study methods and imprecision due to small sample sizes [7].

Over the last decade, a plethora of studies bringing new information on many aspects of LPS have been published in the medical literature. During the same timeframe, four web-based surveys on LPS were conducted through IVF-Worldwide (www.IVF-Worldwide.com). IVF-Worldwide is a comprehensive IVF-focused website linking specialists in IVF centers around the world, providing its members with the ability to communicate and discuss professional topics. IVF-Worldwide also constructs, conducts, and reviews online surveys on a variety of ART-related topics.

The objectives of the current study were to evaluate and compare trends and patterns in the utilization of LPS worldwide using a series of large-scale web-based surveys and to examine whether and to what extent current practices follow the updated evidence-based recommendations available in the literature.

## Materials and Methods

### Survey platform

The IVF-Worldwide platform is non-commercial and has an advisory board of key opinion leaders in the field of IVF. The forum enables access to a large number of clinicians and IVF clinics all over the world possessing a wide spectrum of opinions. Therefore, it is an excellent tool for conducting large-scale surveys. The results of surveys are based on hundreds of thousands of ART cycles conducted in different geographic regions all over the world, seeking the “wisdom of the crowd.“ Thus, surveys conducted by IVF-Worldwide.com can reflect trends and common practices in the industry [8].

Over the past ten years, four surveys conducted related to LPS: *Progesterone support in IVF* (August 2009)[9], *An updated survey on the use of progesterone for luteal phase support in stimulated IVF cycles* (June 2012)[10], *A survey on luteal-phase progesterone support* (January 2018)[11], and *A follow-up survey on luteal-phase progesterone support* (January 2019)[12]. The surveys and their responses were and are still open for public viewing via the link: https://ivf-worldwide.com/survey.html. Invitations to participate in the surveys were emailed to all IVF units registered on IVF-Worldwide.com.

### Quality assurance

To minimize duplicate clinical unit survey reports and eliminate possible false data, we used two software programs (Community Surveys, corejoomla.com, India; BF Survey, Tamlyn Software, Sydney, NSW, Australia) that compared three parameters from the surveyed clinics’ self-reported data with existing clinic data from the IVF-Worldwide website. Methods used were described in previously reported research from the IVF-Worldwide network [13, 14]. These parameters were the unit name, country, and e-mail address. At least two parameters had to match between the survey and the website for the clinical unit data to be included in the study. If two survey responses shared at least two parameters, the duplicate survey results with the later date were discarded.

### Statistical analysis

The analysis was based on the number of IVF cycles performed annually as reported by the units, and not on the number of units in the study. Thus, the relative proportion of answers reflects the total proportion of IVF cycles represented rather than the proportion of individual respondents to the survey questions. We set the maximum number of IVF cycles to 4500 in order to limit the influence of large-scale centers. The surveys were structured as a sequence of multiple-choice questions, in which respondents could select a single answer.

Survey results were calculated by using the formulas described in previously reported research from the IVF-Worldwide network [14–16]. For example, for a question with four possible answers (a, b, c), the following results were calculated:

$$ ^{\prime }{a}^{\prime}\%=\frac{\sum IVF\  cycles\ performed\ annually by\ units\  who\  answered{{}^{\prime }a}^{\prime }}{\sum IVF\  cycles\ performed\ annually\  by\  all\  the\ units\  who\  answered\ the\ survey}\ast 100 $$

$$ ^{\prime }{b}^{\prime}\%=\frac{\sum IVF\  cycles\ performed\ annually by\ units\  who\  answered{{}^{\prime }b}^{\prime }}{\sum IVF\  cycles\ performed\ annually\  by\  all\  the\ units\  who\  answered\ the\ survey}\ast 100 $$

$$ ^{\prime }{c}^{\prime}\%=\frac{\sum IVF\  cycles\ performed\ annually by\ units\  who\  answered^{\prime }c^{\prime }}{\sum IVF\  cycles\ performed\ annually\  by\  all\  the\ units\  who\  answered\ the\ survey}\ast 100 $$

## Results and discussion

The geographic distribution of participating IVF units, with their corresponding estimated annual number of IVF cycles is presented in Table 1. Except for the initial survey, which was the first survey that IVF-Worldwide conducted, and therefore, included a relatively small cohort (97 units, 51,155 annual cycles), subsequent surveys were substantially larger, all including over 270 units representing > 250,000 annual cycles. After conducting more than 25 surveys on the site, our group showed that after reaching a critical mass of 35,000 annual IVF cycles, the statistical results do not change, and they reliably represent the current opinions of the ART community [8]. Although the four surveys were not identical in their content, major issues regarding LPS, such as timing of initiation and withdrawal, formulations and routes of administration, were consistently evaluated and will be presented herein.

**Table 1:** Geographic distribution of survey respondents by survey date

	January 2019		January 2018		June 2012		August 2009	
**Continent**	**Estimated annual IVF cycles**	**No. of IVF units**	**Estimated annual IVF cycles**	**No. of IVF units**	**Estimated annual IVF cycles**	**No. of IVF units**	**Estimated annual IVF cycles**	**No. of IVF units**
**USA & Canada**	35,200	35	44,900	52	26,200	52		
**Central & South America**	9000	27	31,100	59	13,300	46		
**Australia & New Zealand**	14,200	4	15,400	12	17,900	14		
**Asia**	75,600	69	64,900	90	63,300	89		
**Europe**	112,200	120	153,400	200	150,700	185		
**Africa**	19,900	17	12,700	24	13,200	22		
**Total**	**266,100**	**272**	**322,400**	**437**	**284,600**	**408**	**51,155**	**97**

### How is the luteal phase support regimen being chosen?

One of the first questions we asked was: “Which factor is most important to you while deciding which LPS regimen to use?“ While the majority of respondents (71.8 %) used established evidence-based data from the literature, 18.9 % preferred their own personal experience to prescribe LPS, and 5.8 % used published guidelines. Patient-centered issues such as convenience to patients (3.2 %) and cost of medication (0.3 %) received top priority in considerations by a minority of respondents.

### Timing of initiation

As mentioned above, the purpose of LPS in ART is to fill the gap in P secretion from the corpora lutea, driven initially by exogenously administered hCG and later on by embryonic-derived hCG [1]; [17]. Premature administration of P may cause endometrial advancement and embryo-endometrial asynchrony. On the other hand, late administration may not be sufficient to support endometrial development and, therefore, might interfere with endometrial receptivity. Several randomized controlled trials (RCT) evaluated various options of LPS initiation. Three RCTs compared clinical pregnancy rates (CPR) after starting LPS with P on the evening of oocyte retrieval, starting on the evening of embryo transfer. Baruffi, et al. [18] (103 women, 27.4 % vs. 28.8 %), Fanchin, et al. [19] (84 women, 42 % vs. 29 %), and Mochtar, et al. [20] (255 women, 28.1 % vs. 29.1 %) all reported no significant difference in CPR with early versus late start of LPS, respectively. Live birth rate (LBR) was only reported by Mochtar, et al., who found no significant difference between the groups (21.1 % vs. 20.5 %; RR 0.97, 95 % CI 0.60–1.56)[20].

Two RCTs compared the initiation of LPS with P before and after oocyte retrieval. Mochtar, et al. reported no significant difference in LBR (20 % vs. 21.1 %; RR 0.94, 95 % CI 0.58–1.52) or CPR (23.1 % vs. 28.1 %; RR 0.82, 95 % CI 0.54–1.24) with starting LPS either 12 hours before oocyte retrieval or after oocyte retrieval, respectively [20]. In contrast, Sohn, et al. found a significantly lower CPR when LPS was started in the evening of the hCG trigger compared to starting after oocyte retrieval (12.9 % vs. 24.6 %) [21]. Gao, et al. compared starting LPS with P on the day of oocyte retrieval with the day after oocyte retrieval in 233 women and reported no significant difference in LBR (46.6 % vs. 45.7 %, respectively) [22]. Williams, et al., investigated in 126 women starting LPS with P on day 3 or day 6 after oocyte retrieval and found a significantly lower CPR when LPS was started on day 6 compared to day 3 (44.8 % vs. 61.0 %) [23]. From the above studies, it does appear that initiation of LPS too early [21] or too late [23] adversely affects endometrial receptivity and CPR.

In the recent guidelines released by the European Society of Human Reproduction and Embryology (ESHRE), it was recommended to start LPS during the window between the evening of the day of oocyte retrieval and day 3 post oocyte retrieval [6]. The level of recommendation is rather low and is classified as good practice point (GPP), which is the recommended best practice based on the clinical experience of the Guidelines Development Group (GDG).

**Fig. 1:**
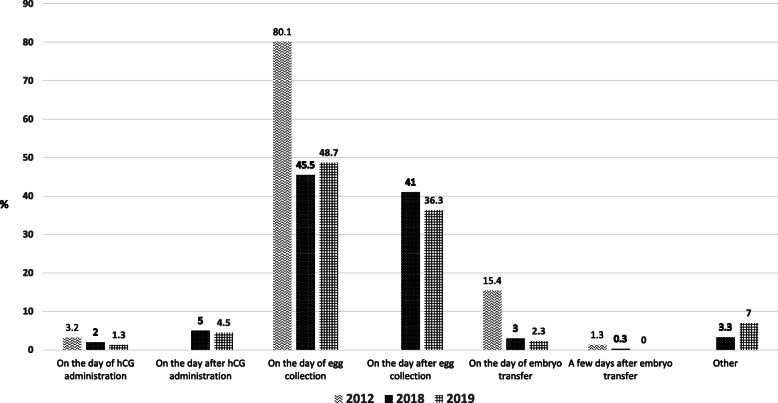
Responses to the survey question: When do you start the regimen you use?

The majority of the survey respondents began LPS administration either on the day of egg collection (48.7 % in the 2019 survey) or on the day after egg collection (36.3 % in the 2019 survey) (Fig. 1). This fits well with the recommendations of the ESHRE guidelines, without major changes in practice evident over the years, except for a drop in the number of cycles in which LPS started on the embryo transfer day (15.4 % in 2012 vs. 2.3 % in 2019).

### Duration

The efficacy of LPS use beyond the point of pregnancy establishment has been questioned by several groups of investigators who compared early cessation of LPS at either the time of positive hCG test or early pregnancy ultrasound versus LPS administration until 7–8 gestational weeks. In the majority of studies, patients were selected for inclusion based on specific criteria, such as normal rising hCG patterns, absence of vaginal bleeding episodes, favorable serum P levels, serum estradiol levels, age, and even normal early (5-week) or routine (6- to 7-week) pregnancy ultrasound [24–30]. Thus, rather than treatment-naïve patients, carefully selected, good-prognosis patients were studied. Despite the bias and heterogeneity introduced by selective patient inclusion criteria, a recent meta-analysis by Watters, et al. [31] summarized the results of 7 randomized trials including 1,627 participants, and found similar LBR, miscarriage, and ongoing pregnancy rates (OPR) with early versus late LPS cessation. The authors concluded that prolonged P supplementation after fresh embryo transfer might be unnecessary. These results are consistent with a previous meta-analysis by Liu, et al. [32].

The recent ESHRE guidelines suggest that administering P for LPS should continue at least until the day of the pregnancy test, and the level of evidence is again rather low (according to the GPP) [6].

**Fig. 2:**
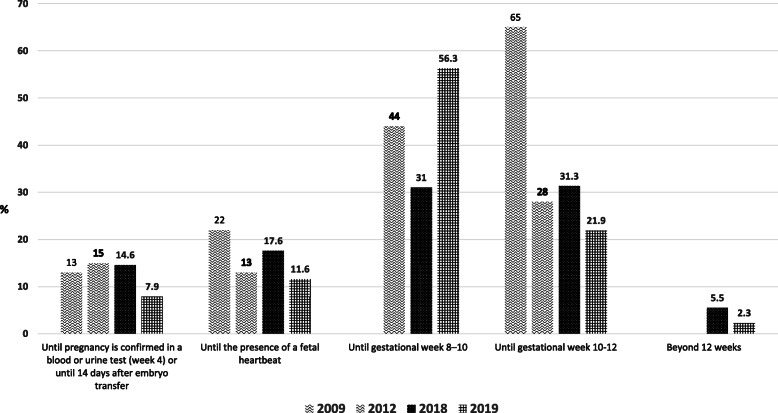
Responses to the survey question: Until how many weeks after embryo transfer do you continue progesterone supplementation if the patient conceives?

While it has been over a decade that most of the above RCTs have been published, and despite the publication of two meta-analyses with similar results and recommendations, the scientific community has been reluctant to adopt early cessation of LPS. This can be clearly evidenced by taking a close look at the results of our series of LPS surveys, which reveals that the majority (> 60 %) of clinicians worldwide administer LPS until 8 gestational weeks and beyond, without any significant change in trends over the last decade (Fig. 2). In the 2018 survey 72 % of respondents administered LPS until 8–10 gestational weeks, and the most recent 2019 survey revealed that 65 % of the respondents continued LPS until 10–12 gestational weeks. Another survey conducted in the United Kingdom’s ART community yielded similar results [33]. These findings represent the perception that the quality of data regarding early cessation of LPS is weak and insufficient to recommend a change in practice. Clinicians need to obtain a high level of confidence of “primum non nocere” or “first do no harm” before adopting routine early withdrawal of LPS.

### Route of administration

#### Intra‐muscular (IM)

Because P is water-insoluble, P-in-oil mandates IM administration, which avoids the hepatic “first pass” associated with oral administration. IM-P has obvious drawbacks, which limit convenience to patients and adherence to treatment, because administration is notoriously painful, IM-P is a source of allergic reactions, and it carries a risk of sterile abscesses formation. Consequently, IM-P, once considered the gold standard route for P administration, has been gradually replaced by other formulations and routes of administration, the vaginal route being the most popular.

In a recent systematic review and meta-analysis of RCTs of vaginal vs. IM-P for LPS in ART, IM-P showed similar results in CPRs, OPRs, miscarriages, and LBRs [34]. Similar results were obtained in the updated Cochrane meta-analysis [7].

According to the recent ESHRE guidelines, any (non-oral) administration route for natural P as an LPS can be used (level of evidence: GPP).

According to the surveys on LPS, the use of IM-P decreased from 13 % in 2009 to 4.6 % in 2019. Among respondents who used IM-P, 74.8 % used daily P-in-oil, 17.9 % use long-acting P formulations, and 7.3 % use a different interval of administration or formulation.

#### Subcutaneous progesterone (SC)

The search for practical options that avoid painful IM-P injections while retaining its reliable efficacy led to the development of an aqueous P preparation that can be self-administered SC. An individual patient data (IPD) meta-analysis of available phase III trials compared SC-P to vaginal P for LPS in IVF (2 RCTs, 1435 women) [35]. The administration of SC-P versus vaginal P had no impact on ongoing pregnancy likelihood (OR = 0.865, 95 % CI 0.694 to 1.077; P = NS), live birth likelihood (OR = 0.889, 95 % CI 0.714 to 1.106; P = NS) or ovarian hyperstimulation syndrome (OHSS) risk (OR = 0.995, 95 % CI 0.565 to 1.754; P = NS).

Because SC-P has become commercially available only recently, it is being introduced gradually and cautiously into clinical practice. Only 0.4 % of respondents in the 2018 and 2019 surveys use SC-P as their first line of treatment. However, this method had the highest pick in the combination of drugs, indicating that some clinicians tended to rely on IM/SC administration as a safety net.

#### Vaginal progesterone

The unique properties of direct P transport from the vagina to the uterus and the uterine first pass effect [36, 37] have led to the development and large-scale application of vaginal P products for LPS. A comparison between vaginal/rectal and IM-P routes in the updated Cochrane meta-analysis reported no difference in LBR (4 RCTs, 1,222 patients, OR 1.17, 95 % CI 0.91 to 1.51) [7]. A recent systemic review of 18 trials comparing 4 vaginal P products have shown that there are no significant differences in efficacy or safety between the products [38].

**Fig. 3:**
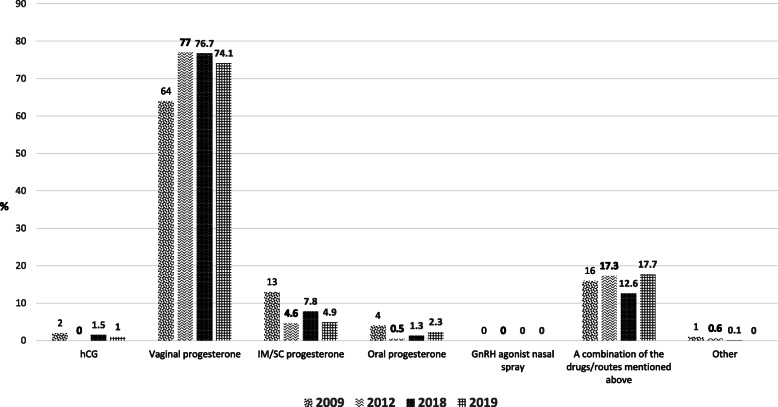
Responses to the survey question: In the majority of the cases, what is your treatment agent/route of choice to support the luteal phase?

Throughout the surveys, the vast majority of respondents preferred the vaginal route of delivery (Fig. 3), and its utilization rose from 64 % of cycles in 2009 to 74.1 % in 2019. With regard to the preferred vaginal formulation/product, in 2018, 46.7 % preferred vaginal tablets, 25.9 % vaginal gel, 13.8 % vaginal suppositories, 10 % vaginal pessaries, 2 % other, and 1.6 % did not use vaginal P, consistent with the results obtained in the 2012 survey.

#### Oral preparations: micronized progesterone and dydrogesterone

The development of the micronization process has enabled much improved absorption of oral P. However, P cannot be administered orally in ART due to intense hepatic metabolism during the first liver pass, which cannot be overcome by simply increasing the doses of P administered, since it produces a degree of somnolence unacceptable to most patients. Oral micronized P was used for LPS in the early days of IVF, with poor results until the end of the 1990s [39–41]. Serum levels of P are too low after oral administration to provide adequate endometrial support [42], and oral micronized P failed to induce the secretory transformation of the endometrium in patients with primary ovarian insufficiency (POI) [43, 44]. Therefore, since oral micronized P cannot be relied upon for LPS, its use has been abandoned, and it was not recommended for LPS according to the recent ESHRE guidelines [6].

Dydrogesterone (DG) is a retroprogesterone, a biologically active metabolite of P, with good oral bioavailability that may overcome the problem of massive metabolism of micronized P. Dydrogesterone has been used to treat a variety of conditions related to P deficiency since the 1960s and has recently gained renewed interest following its approval for LPS in ART. A recent meta-analysis comparing the use of oral DG and vaginal P for LPS indicated that DG provided at least similar results to vaginal P capsules on live birth/ongoing pregnancy (RR = 1.08, 95 % CI = 0.92–1.26, I2 = 29 %, 8 RCTs, 3,386 women) and CPR (RR 1.10, 95 % CI 0.95 to 1.27; I2 = 43 %; 9 RCTs; 4,061 women) [45]. An RCT published subsequent to the above meta-analysis compared oral DG with vaginal P gel and also reported no significant difference in LBR [34.4 % (170/494) vs. 32.5 % (159/489); 1034 women] [46].

The recent ESHRE guidelines state that “DG is probably recommended for LPS” with a moderate level of evidence [6].

In 2009, oral P was prescribed in 2 % of cycles. This number decreased to 0.5 % in 2012, rising to 1.3 % in 2018 and again up to 2.3 % in 2019. The decline in oral P use seen between 2009 and 2012 probably reflects the abandonment of oral micronized P use for LPS, and the recent increments that were noted most likely represent the introduction of oral DG for LPS.

In the recent 2019 survey, when asking clinicians if they were aware of recently published studies in favor of the oral route for LPS, 69.4 % replied positively, but they would like to see more evidence, 16 % replied that the data was sufficient, and 14.6 % replied that they were not aware of published studies.

Among those who do prescribe oral P for LPS, we asked which drug they preferred. A total of 63.5 % selected DG, 35.7 % chose micronized P, 0.8 % preferred medroxyprogesterone acetate, and none selected norethisterone.

In the recent 2019 survey, we asked: “If all P formulations were found to yield the same LBRs, which route would you prefer to use?“ A total of 62.2 % selected the oral route, 30.1 % chose vaginal, 5.2 % opted for subcutaneous, 1.3 % preferred rectal, none selected the IM route, and 3.2 % had no preference. Moreover, when we asked what women would prefer, 85.9 % thought they would prefer the oral route.

#### Drug combinations for LPS

A consistent proportion of clinicians prefer a combination of products and routes of administration for LPS (16 % in 2009 and 17.7 % in 2019). In a follow-up question on combination of P treatments, 36 % used vaginal and IM/SC injections; 27.4 % used vaginal and oral; 1.5 % used IM/SC and oral; 0.9 % used vaginal, oral and IM/SC; 2.1 % used other combinations; and 31.8 % did not use combinations.

#### Human chorionic gonadotropin

The ability of the CL to be rescued by exogenously administered hCG made hCG standard care for LPS in the early days of IVF [4]. In the Cochrane meta-analysis, higher live birth/ongoing pregnancy rates were achieved with hCG for LPS, compared to placebo/no treatment (3 RCTs, OR 1.76, 95 % CI 1.08–2.86, 527 women) [7]. The drawback of using hCG for LPS lies in its potential for increasing rates of OHSS when compared with other interventions or no treatment [7]. When compared to progesterone, hCG alone for LPS or a combination of P and hCG showed comparable LBRs/OPRs (5 RCTs, OR 0.95, 95 % CI 0.65–1.38, 833 women). Progesterone, however, was associated with lower OHSS rates than hCG with or without P (5 RCTs, OR 0.46, 95 % CI 0.30–0.71, 1293 women) [7]. Therefore, although its efficacy has been well proven, the use of hCG for LPS in stimulated cycles has dramatically declined due to serious safety issues.

According to the ESHRE guidelines, in hCG-triggered ovarian stimulation cycles, hCG as an LPS in standard dosages of 1500 IU is probably not recommended, although the quality of evidence is low [6].

A very low proportion of our survey respondents used hCG as their predominant method for LPS: 4 % in 2009 and only 1 % in 2019.

#### Estradiol

The inclusion of estradiol (E2) in LPS regimens is debatable. While some investigators showed value in adding E2 [47–49], others did not [50–52]. The Cochrane meta-analysis concluded that there is no benefit in adding E2 to P for LPS (9 RCT, OR 1.12, 95 % CI 0.91–1.38, 1651 women) [7]. According to the recent ESHRE guidelines, the addition of E2 to P for LPS is probably not recommended, although the quality of evidence is low [6].

In the 2018 survey, when we asked about the inclusion of E2 in LPS regimens, 16.6 % of respondents answered “yes, always”; 45.3 % answered “yes, but only in selected cases”; and 38.1 % answered “no.” This variety of opinions settles well with the conflicting data.

#### GnRH agonist

The possibility of supporting the luteal phase of ART cycles solely by repeated administration of an intranasal GnRH agonist has been demonstrated in a small randomized trial by Pirard, et al. [53] and more recently in a large retrospective study by Bar Hava, et al. [54]This treatment modality has not been investigated through large RCTs, and concerns have been raised regarding its safety [55]. Additional regimens for the inclusion of GnRH agonists in LPS regimens, such as a single GnRH agonist bolus in the mid-luteal phase or the addition of a GnRH agonist to P supplementation have also been suggested [6].

In the recent ESHRE guidelines, it was recommended that all LPS regimens that include a GnRH agonist in hCG-triggered cycles, including repeated GnRH agonist injections, alone or in addition to P and a GnRH agonist bolus in addition to P, can only be used in the context of a clinical trial [6].

In our most recent surveys (2018 and 2019), we only asked about the use of the GnRH agonist nasal spray, and none of the respondents in both surveys was using this method as their predominant approach for LPS.

#### Personalized luteal phase support

Improvement in and individualization of COS regimens have been the subject of intensive research in the field of ART, and patient-tailored COS has now become the state of the art. In contrast, the luteal phase of fresh IVF cycles has received little attention, and studies focusing on luteal phase characteristics following hCG triggering are scarce and have yielded conflicting results [56–59]. Neither the updated Cochrane meta-analysis on LPS [7], nor the ESHRE guidelines [6] have included recommendations regarding patient monitoring during the luteal phase.

In the 2018 and 2019 surveys, we included a series of questions to evaluate the growing interest in personalized LPS. When determining how LPS regimens are assigned to patients, 55.4 % of respondents individualized LPS regimens (according to patients’ ovarian response, stimulation protocol, age, BMI, etc.) and 42.1 % use the same LPS regimen for all patients. When patients fail in their first cycle, the majority of respondents (77.2 %) will leave the LPS unchanged in the next cycle, and only a minority will change the P medication (9.8 %) or add another P product to the regimen (9.4 %).

When asked about routine measurements of P levels during the luteal phase, 19.9 % of respondents replied that they measured as standard practice, as opposed to 66.8 % who said they did not and 13.3 % who measured P levels only in individually selected cases. Among those who routinely measured P during the luteal phase, 47.4 % may have increases the dose, 29.6 % may have added additional P to the regimen, 5.5 % may have lowered the dose, and 17.5 % did not modify treatment, retaining the data only for the future.

It seems very likely that one of the next leaps in reproductive medicine will be the individualization of LPS regimens in order to optimize medical treatment and increase IVF success rates. Intensive research on the endocrinology of the luteal phase in stimulated cycles is necessary in order to enable a patient-tailored approach of LPS. Although the medical community has been lagging in comprehending the opportunities that lie in big data and machine learning, there is now a growing understanding that we now do have the tools to personalize medical treatment [60].

## Conclusions

Over the last ten years, we have conducted four web-based surveys on the practice of LPS. A quick and simple search on Pubmed of the term “luteal phase support” yielded 1879 papers published between January 2009 and May 2020. Despite the extensive research and numerous publications that we have witnessed over the past decade, there is yet no single accepted protocol for LPS, and meta-analyses and guidelines for clinicians are based on evidence of low quality for the majority of topics discussed. Therefore, it is interesting to observe the gap between science and practice, as evidenced through our web-based surveys. Our surveys, which are clinical practice oriented and had the highest outreach among IVF-Worldwide surveys, representing hundreds of clinics and hundreds of thousands of ART cycles conducted worldwide, enabled us to gain insights on the views and opinions of the ART community on the use of P for LPS.

The majority of clinicians initiated LPS treatment on the day of egg collection or on the day after, and this practice has not changed with time. Regarding the duration of treatment, despite several RCTs and a recent meta-analysis suggesting that LPS can be stopped at very early stages of pregnancy, as also recommended in the ESHRE guidelines, the majority of clinicians worldwide continue LPS until 8–12 gestational weeks, without a change in the trend during the last decade.

The vaginal route has always been the preferred route of administration by our survey respondents, across all surveys. Over the years, we have noticed a gradual increase in the use of vaginal P for LPS, at the expense IM-P administration frequency, which decreased concomitantly. An intriguing point is the fact that the percentage of oral P utilization remained negligible, without much change over a period of ten years, despite novel research results and a meta-analysis regarding the efficacy of oral DG, and despite physician perceptions that the oral route would be the preferred route of administration by patients. The extent of utilization of SC P for LPS is also very low, despite its practical advantages over IM-P. The use of hCG as a sole LPS agent is rare and limited by its inherent risk for OHSS. Both E2 and GnRH agonists are rarely used for LPS.

If we set our eyes on the future, the majority of clinicians worldwide would like to base their practices and recommendations on high quality evidence-based research and professional guidelines. Only a minority of respondents use their personal experience as the major force influencing their clinical judgement. There is also growing demand and understanding that the perception of “one size fits all” for LPS might be outdated. Enhancing clinicians’ understanding of the luteal phase following COS using different modes of ovulation triggering may enable them to individualize LPS to improve convenience to patients, compliance, and success rates. We believe that this subject will have a considerable part in the following phase of this endeavor.

The major strength of our study is the large number of respondents who participated following the initial survey, and that number remained stable over the years, representing over 250,000 IVF cycles performed annually world-wide. We have previously shown that this number is sufficiently high to reliably represent the current opinions of the ART community[8]. Limitations in our study include the fact that the 4 sequential survey questionnaires were not identical and evolved over time. Moreover, as in all surveys, results are subject to reporting bias, and even though the influence of each respondent was limited, it still needs to be considered as a limitation.

In conclusion, advances in ART have made a huge impact on humankind around the globe. Over the last four decades, we have seen major advances and breakthroughs in the individualization of COS and in laboratory techniques. The results of our surveys emphasize that now is the time for the luteal phase to be studied in depth and for LPS to become individualized and tailored to each patient.

## Data Availability

All surveys and survey results are available online (https://ivf-worldwide.com/survey.html).

## Abbreviations

CL: Corpus luteum
ART: Assisted reproductive technology
LPS: Luteal phase support
IVF: In vitro fertilization
COS: Controlled ovarian stimulation
ICSI: Intracytoplasmic sperm injection
RCT: Randomized controlled trials
CPR: Clinical pregnancy rates
LBR: Live birth rate
GPP: Good practice point
GDG: Guidelines development group
IM: Intra-muscular
SC: Subcutaneous progesterone
IPD: Individual patient data
P: Progesterone
OHSS: Ovarian hyperstimulation syndrome
POI: Primary ovarian insufficiency
DG: Dydrogesterone
ESHRE: European society of human reproduction and embryology
hCG: Human chorionic gonadotropin
E2: estradiol
GnRH: Gonadotropin-releasing hormone
